# Case studies associated with the 10 major geodiversity-related topics

**DOI:** 10.1098/rsta.2023.0055

**Published:** 2024-04-01

**Authors:** Murray Gray

**Affiliations:** School of Geography, Queen Mary University of London, Mile End Road, London E1 4NS, UK

**Keywords:** geodiversity, geosystem services, geoconservation, geoheritage, geotourism, sustainability

## Abstract

This paper outlines the 10 major topics related to geodiversity that have emerged since the concept was first introduced in 1993, 30 years ago. After a short introduction, each of the 10 topics is then illustrated by a relevant case study. The 10 topics (italics) and their case studies (bold) are as follows: 1. *Celebrating*, **International Geodiversity Day**; 2. *Measurement/Assessment*, **Potential role of remote sensing**; 3. *Natural Capital and Geosystem Services*, **Coastal geosystem services**; 4. *Biodiversity*, **Mangue de Pedra, Brazil**; 5. *Geomaterials*, **The circular economy**; 6. *Geotourism*, **World's top geotourism sites?**; 7. *Geoheritage*, **Landscape restoration**; 8. *National Geoconservation*, **Trump golf course and an SSSI, Scotland**; 9. *World Heritage Sites and Global Geoparks*, **Azores Global Geopark, Portugal**; 10. *Sustainability*, **Xitle Volcano, Mexico City**. It is concluded that, given the way in which geodiversity has developed as a concept, leading to new insights and avenues of research and advancing our understanding of the world since its first use, it clearly now constitutes a significant, geoscientific paradigm.

This article is part of the Theo Murphy meeting issue ‘Geodiversity for science and society’.

## 1. Introduction

The concept of geodiversity, as currently understood, was introduced in 1993, following the adoption of the Convention on Biological Diversity at the Rio Earth Summit. Many geoscientists realized that they also study diverse phenomena that are also often threatened by human actions, and so the word ‘geodiversity’ seemed entirely appropriate. Its early progression took place in Tasmania (e.g. [1–4]) but most of the criticism of the concept has come from mainland Australia (e.g. [5–7]; see [8]). Despite this scepticism, geodiversity is now a widely accepted international concept as evidenced by the sections of this paper, its reference list and the many research groups working on geodiversity topics around the world. Geodiversity has been defined as ‘the natural range (diversity) of geological (rocks, minerals, fossils), geomorphological (landforms, topography, physical processes), soil and hydrological features’ [9]. Boothroyd & McHenry [10] found that 88% of the 300 papers defining geodiversity published between 1993 and 2019 supported this definition or variants of it. Since geodiversity comprises geology, geomorphology, pedology and hydrology, it is more appropriate to regard it as a shortened form of ‘geoscientific diversity’ rather than ‘geological diversity’ as originally conceived.

Since its first use, geodiversity has snowballed as a concept and has led to many new insights, new avenues of research and new results. The culmination of these efforts was the pronouncement by UNESCO that 6 October each year would be International Geodiversity Day. This paper outlines 10 case studies of diverse styles, contents and lengths related to the 10 major topics associated with geodiversity.

## 2. Celebrating

Nature comprises both living and non-living elements though in the academic world, public forums and the media it is the living world that dominates. Television programmes, such as the huge number of Sir David Attenborough's wildlife series, are extremely popular, and their main aim is to celebrate life on planet Earth. While there have been geoscience programmes, such as those presented by Prof. Iain Stewart, they do not attract the same number of viewers and their aim is to describe and explain the geological wonders of the world rather than to celebrate them. The same is true of the numerous Earth science textbooks whose main aim is also to describe and explain. Yet we live on a magnificent and abiotically diverse planet in terms of the endogenic and exogenic processes operating on it and the materials and forms produced. Human societies have benefitted from this geodiversity for many millennia and in a huge number of ways, starting, for example, by living in caves or hewn shelters, making increasingly sophisticated stone tools, or creating ‘buffalo jumps’ where herds were driven to their death over suitable cliffs. And today our modern societies could not exist without the planet's geodiversity whether in the fields of, for example, resources, culture or environmental quality. These ‘geosystem services’ are outlined in §4 below and described in detail by Gray [9], but it is clear that geodiversity brings huge benefits to society, and appreciating our planet's abiotic diversity can enrich our lives and deserves to be celebrated.

### (a) Case study: International Geodiversity Day

The first International Geodiversity Day (IGD) was held on 6 October 2022 and resulted from an 18-month campaign and process for recognition by UNESCO [11]. The idea of the Day emerged on 22 May 2020 when both Zbigniew Zwolinski and I independently noted that this was International Biodiversity Day, and both thought that achieving an IGD would be beneficial to our subject. Coincidentally, we both shared this thought with José Brilha who agreed with this idea. A few days later, the first Oxford Geoheritage Virtual Conference, principally organized by Jack Matthews, was held online and attended by over 600 delegates from more than 60 countries. At the end of it, a declaration was agreed supporting the establishment of an IGD and recognizing the need to increase public understanding and awareness of geodiversity, its interrelation with other areas of conservation and its role in achieving many of the UN's Sustainable Development Goals (SDGs; see §11 below). Zwolinski *et al*. [11] describe the process and timeline involved towards UNESCO's General Conference in November 2021. On the first IGD, the Director General of UNESCO, Ms Audrey Azouley, released a statement calling on the international community to use this new event to ‘view familiar landscapes through fresh eyes … (and) celebrate the close relationship between biodiversity, geodiversity, culture and history, raising awareness of ‘nature's stage’. Celebration of the role geodiversity plays in so many ways is, therefore, a key aim of the IGD.

**Figure 1.**
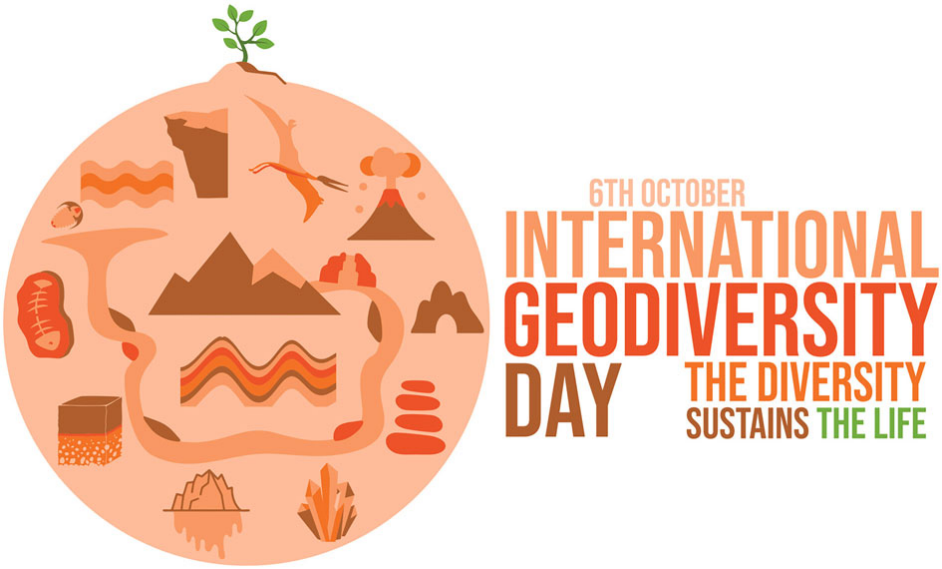
The International Geodiversity Day logo. (Online version in colour.)

The 6 October was chosen for IGD as (i) October was the month of publication of the first known document to use ‘geodiversity’ with the meaning we use today, written by Chris Sharples in Tasmania in October 1993 [1], (ii) it allows for field activities in reasonable weather in both hemispheres and (iii) 6 October was the first date in October free of other international days. According to analysis by Jack Matthews, on the first IGD in 2022, 249 events were registered on the IGD website (www.geodiversityday.org) but many events were not registered. India held the most events (52) most being organized by INTACH (the Indian National Trust for Art and Cultural Heritage). Next came Portugal (30), United Kingdom (29), Spain (26), Romania (21) and Italy (11). Figure 1 shows the logo for the IGD.

## 3. Measurement/assessment

The quantitative measurement or qualitative assessment of geodiversity is a very active field of research (e.g. [12–16]). Most of the studies that have been carried out have been based on published, small-scale maps of geology, topography, soils, drainage, etc. or sometimes on remote sensing images, and have been carried out on scales ranging from regions to continents [17]. The quantitative method generally involves placing a rectilinear grid over the study area and then counting the number of occurrences of, for example, rock types, soil types, etc. within each grid square. Finally, a geodiversity map is produced by combining, and sometimes weighting, the individual counts. I am on record as saying that this method undermines ‘the full richness of geodiversity, one of its most important characteristics. What is being measured is not total geodiversity since small-scale features/subtle differences will be missed using small-scale maps…neither maps nor remote sensing can identify/interpret dynamic processes, functions or origins…’ [18, p. 609] and ‘As a result, many of these studies oversimplify geodiversity to a quite extraordinary extent and, in many ways, are the antithesis of geodiversity’ [19, p. 15]. This is not to say that geodiversity measurement is not relevant in certain circumstances, but we need to improve our measurement methods. One of these is through remote sensing as illustrated by the following case study.

### (a) Case study: potential role of remote sensing for Earth observation of geodiversity

Earth observation (EO) is the gathering of information about the physical, chemical and biological systems of the planet via remote-sensing technologies, supplemented by surveying techniques, the purpose being to monitor and assess changes in natural and built environments. In a series of three papers, Lausch *et al*. [20–22] describe the current and potential for remote sensing to assess geodiversity and link it with biodiversity. In the first of these, Lausch *et al*. [20] describe how remote sensors mounted on drones, microlights, helicopters, aircraft or satellites can detect several surface and soil mineralogical characteristics which can be linked to biodiversity. This includes using visible, near infrared and short-wave thermal infrared sensors to detect clay, sand, carbonate, silicate, sulfate or iron content. Other studies have demonstrated how sensors can detect clay minerals (illite, kaolinite), sulfate minerals (alunite), carbonates (dolomite, calcite), iron oxides (goethite, haematite) and silica. There is future potential to distinguish between quartz/feldspar and sheet or chain silicates. Lausch *et al*. [20, p. 9] conclude that ‘this type of information can thus be used to monitor and quantify the surface mineralogy that has an impact on biodiversity, from local to global scales’. Sensors are also able to detect bare, topsoil variables such as soil texture, soil organic carbon, soil moisture, iron content, soil salinity and carbonates. In their second paper, Lausch *et al*. [21] examine the role of current and future LIDAR, RADAR, multispectral and hyperspectral sensor technologies to monitor geomorphology, processes and hazards and their relationship to biodiversity and nature conservation. The techniques enable topography/digital elevation models (DEMs) to be constructed, sometimes to high resolution with LIDAR. Examples are given of the monitoring of aeolian landforms, fluvial landforms (including flood events, flood risks, fluvial and tidal channel migration, and riverbank retreat) and coastal landforms. They conclude that ‘Geodiversity controls biodiversity… (it) is the promoting, controlling, regulating and limiting factor, as well as the most important for landscape processes…implying that a successful conservation of biodiversity primarily entails the conservation of geodiversity’. In the final paper, Lausch *et al*. [22] extend this geomorphological approach by examining in detail the role of remote sensing in describing and monitoring geomorphodiversity and its relationship to biodiversity.

Other authors have also researched the role of remote sensing in mapping elements of geodiversity and the link to biodiversity. For example, Zarnetske *et al.* [23] used remotely sensed data to examine the relationship between topographic elevation and tree biodiversity across the western USA. They believe (p. 549) that ‘Interdisciplinary research teams spanning biodiversity, geoscience and remote sensing are well poised to advance understanding of biodiversity–geodiversity relationships across scales and guide the conservation of nature’. They also make the point that the field of remote sensing is advancing rapidly with technical advances bringing new sensors and new missions. A further example, this time mapping offshore geodiversity, is Dolan *et al.*'s [24] description of the work of the Norwegian mapping agencies to use acoustic remote sensing (multibeam bathymetry, backscatter, water-column data and sub-bottom profile data) to map parts of the offshore areas around Svalbard and deeper parts of the Norwegian Sea. Finally, in the restoration case study in §8 below, the example is given of remote sensing being used as a cheaper way than field survey of identifying where drainage channels remain to be blocked in order to restore wetland bog landscapes and reduce flood risk.

## 4. Natural capital and geosystem services

Several types of capital exist, including financial capital, social capital, intellectual capital and natural capital. A common definition of natural capital is that it is ‘the world's stocks of natural assets which include geology, soil, air, water and all living things’. As already explained, these natural capital assets have been used by human societies for millennia through what is commonly called the ‘ecosystem services’ approach. However, the current implementation of this approach is very biocentric and often excludes services associated with geodiversity, either consciously or unconsciously [25]. For this reason, the term ‘geosystem services’ has been used by some authors (e.g. [9,26–28]). Gray [9] describes 25 major services which, as a group, are all derived from the planet's geodiversity. These are shown in table 1 using the classification from the Millennium Ecosystem Assessment [29] with the addition of ‘knowledge services’. The next four sections of this paper describe some of these services and a case study is given below.

**Table 1.** The 25 major geosystem services with examples from the coastline.

geosystem services	coastal examples
regulating	
1. atmospheric and oceanic processes	ocean and tidal currents; wave action
2. terrestrial processes	carbon sequestration in saltmarshes
3. flood regulation	barrier islands; sand dunes
4. water quality regulation	dune sands as natural filters
supporting	
5. soil processes	soil development on coastal plains
6. habitat provision	mangroves; mudflats for wading birds
7. land and water as a platform	agriculture; golf courses; coastal cities, towns, villages
8. burial and storage	offshore oil and gas; carbon capture and storage
provisioning	
9. food and drink	sea salt; freshwater from desalination
10. nutrients and minerals	minerals from soil via crops for healthy growth
11. energy resources	offshore hydrocarbons; tidal and wind power
12. construction materials	seabed sand and gravel; coastal quarries
13. industrial minerals	phosphates from seabird guano
14. ornamental products	coastal placers; beach finds of jet, amber, etc.
15. fossils	fossils in coastal exposures
cultural	
16. environmental quality	coastal landscapes for mental and physical health
17. geotourism and leisure	beaches; coastal scenery; swimming; surfing
18. cultural, spiritual and historic meanings	French D-Day beaches; coastal folklore
19. artistic inspiration	geology in sculpture, literature, music poetry, etc.
20. social development	local geological societies; coastal field trips
knowledge	
21. Earth history/geoheritage	coastal geoheritage; coastal World Heritage Sites and geoparks
22. history of research	understanding unconformities and igneous rocks
23. environmental monitoring	sea-level change; wave and storm data; acidification
24. geoforensics	origin of distinctive beach sands
25. education and employment	professional training; employment in geoparks

### (a) Case study: coastal geosystem services

**Figure 2.**
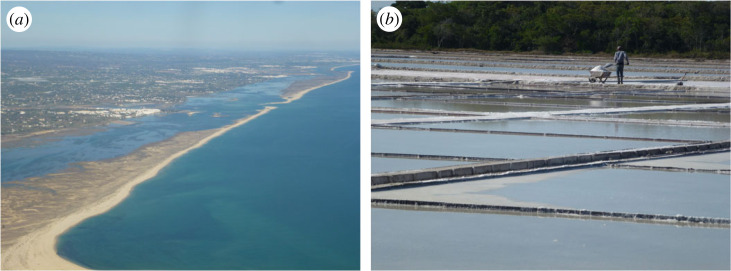
(*a*) Barrier islands, Ria Formosa, Algarve, Portugal. (*b*) Harvesting sea salt, Armação dos Búzios, Brazil. (Online version in colour.)

The coast is a very appropriate place to illustrate the diversity of geosystem services (table 1). For example, in terms of Regulating Services, salt marshes are important for carbon sequestration. According to the UK's Wildlife Trusts, a hectare of saltmarsh can capture two tonnes of carbon a year and lock it into sediments for centuries. Also, alongside barrier islands (figure 2*a*) and sand dunes, saltmarshes act as protectors of coasts from flooding and erosion. Saltmarshes and mudflats are also important habitats for plant, insect and bird life in the Supporting Services category, for example for wading birds, waterfowl and mangroves (see §5 below). The coastline also provides a platform on which many of the world's great cities and towns have been constructed and continue to expand. Offshore areas have been explored for oil and gas resources stored in reservoir rocks with the potential to be future carbon capture and storage sites. Offshore oil and gas themselves can also be included in the Provisioning Services category, along with renewable energy sources of wind, wave and tidal power. Also, under provisioning, are sea salt supplies (figure 2*b*), water desalination and submarine sand supplies. Under Cultural Services, the attraction of coastal views for housing sites are well known and can add significantly to the value of housing. Coastal environments also count as important for mental and physical health and well-being [30], while beaches are by far the world's most important geotourism destinations. Related to this are important coastal leisure activities including links golf (see §9 below) and sea swimming, surfing and sailing, though some of these activities bring safety issues. Coasts are also locations for folklore, historical associations and a sense of place, as well as being a source of inspiration for artists, poets, composers and writers. Finally, the coastline provides us with Knowledge Services, including important geoheritage sites (see §§8–11 below) because of the degree of exposure of the rocks, sediments and their included fossils.

## 5. Biodiversity

The two-way relationship between biological and geological systems has a long history going back billions of years, from the first development of life in oceanic black smokers, through the colonization of barren land surfaces of weathered rock (soil) able to provide nutrients for plant growth and on to the first terrestrial plant life from the Middle Ordovician around 430 Ma. Valentine & Moores [31] and Hallam [32] were the first to suggest a relationship between the development of biodiversity and plate tectonics, and this has been confirmed by more recent studies (e.g. [33–35]). And the study of modern relationships between the two also has a long history going back to Alexander von Humboldt's studies of the relationship between vegetation, altitude and aspect and formalized as ‘biogeomorphology’ by Viles [36].

More recently, the influence of geodiversity on biodiversity has become a very active field of research and is increasingly involving ecologists and multidisciplinary teams (e.g. [20–23,37–46]). The reason for this is that it is now recognized that biodiversity is strongly influenced by the physical environment [47]. In turn, this means that geodiversity has the potential to be a predictor of biodiversity so that geodiversity data can be used even when biodiversity data are absent or too expensive to collect. Furthermore, because of the strong links demonstrated in many studies and at all scales, it has been proposed that biodiversity conservation might best be achieved by conserving geodiversity. This idea [48–50] has become known as the ‘Conserving Nature's Stage’ (CNS) approach, using the theatrical metaphor of the wildlife being the actors and geodiversity being the stage. A special issue of the journal *Conservation Biology* [51] was devoted to this theme. This approach is likely to be particularly important at times of climate and environmental change, allowing wildlife to migrate to suitable physical habitats and niches [52] and ensuring that landscapes are more resilient to future change.

### (a) Case study: the Mangue de Pedra aquifer and mangrove ecosystem, Brazil

**Figure 3.**
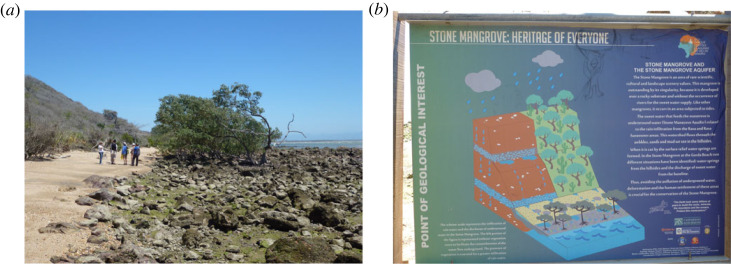
(*a*) The Mangue de Pedra mangrove and beach, Armação dos Búzios, Brazil. (*b*) Information panel in English at the Mangue de Pedra (Stone Mangrove) site. (Online version in colour.)

The Mangue de Pedra mangrove, or Stone Mangrove, is located at Armação dos Búzios, 135 km east of Rio de Janeiro in Brazil. Unlike most of the world's mangroves, this one is unusual as it occurs not in muddy, coastal sediments but on a boulder and sand beach comprised chalcedony breccias (figure 3*a*). Furthermore, it occurs where there is no fluvial input but instead is fed by the Mangue de Pedra aquifer draining directly onto the beach or from springs on the slope behind the beach (figure 3*b*). This mangrove is, therefore, dependent on groundwater flow rather than being rooted in coastal mud. The local substrate and role of fault structures affecting the aquifer–mangrove ecosystem were investigated by Albuquerque *et al*. [53] using hydrochemical analyses and geophysical surveys, including electrical resistivity tomography and ground penetrating radar. The research demonstrated the importance of local faults in creating pathways for groundwater flow and traps or barriers within the aquifer which channel less-saline waters. The result is a close relationship between geodiversity and biodiversity and a ‘unique ecosystem’ not normally occurring on beaches on open sea shorelines.

## 6. Geomaterials

**Figure 4.**
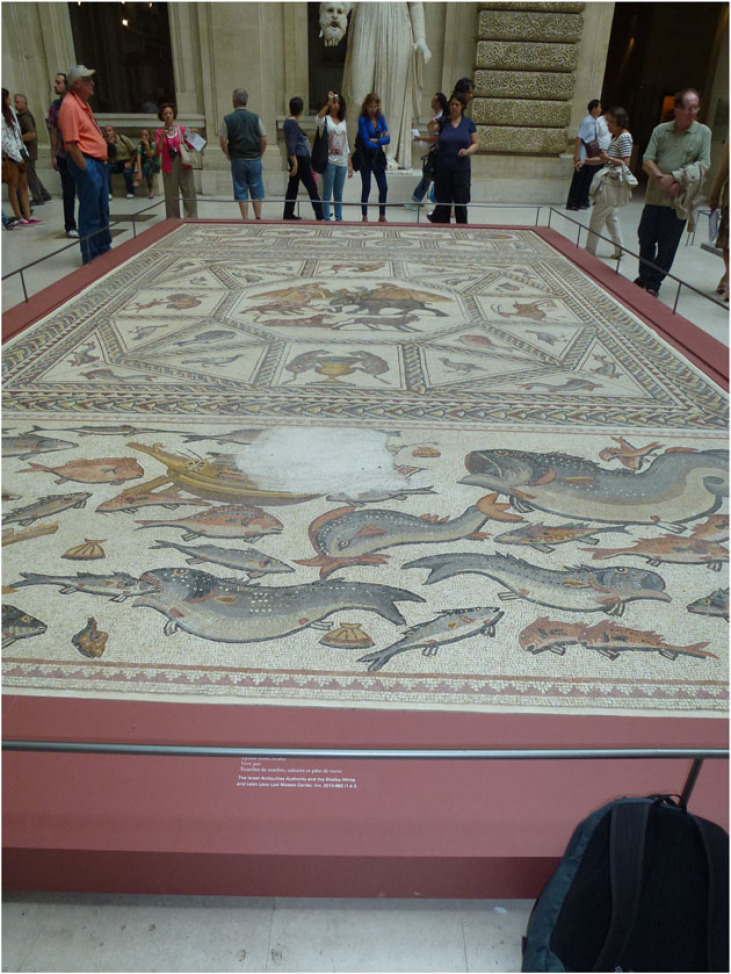
Roman mosaic from Lod, Israel, on display at the Louvre Museum Paris, 2013. (Online version in colour.)

As already referred to in §2, for many millennia human civilizations have made excellent use of the diversity of the planet's physical resources. During the Palaeolithic, the search for geomaterials to make hand axes and arrow heads led them to flint, obsidian, quartzite and other hard lithologies that split into sharp edges. They also used stone to construct crude shelters which, like tools, became more sophisticated over time. The Romans were experts in using different coloured stones to create mosaics for their walls and floors (figure 4). Metals were also discovered and brought new advances in tools and weapons during the Bronze and Iron Ages. Moving forward to the present day, our modern societies simply could not function without exploiting the planet's geodiversity, whether for our telecommunications, our transport systems, our domestic and commercial lighting and heating systems or a thousand other uses in our everyday lives. The perfect example is the typical smartphone which contains over 75% of the non-radioactive elements in the Periodic Table, all of which have been extracted from the Earth's crust and all of which play different roles within the phone [54,55]. So when we hold a smartphone, we literally have geodiversity in our hands!

Metals, energy minerals and gemstones are high-value geomaterials but in terms of weight represent only a small percentage of the total geomaterials extracted from the lithosphere. It is the bulk minerals such as sand, gravel and crushed stone that constitute the major quantity of geomaterials used today. And, because of their weight and cost of transport, they are usually used close to their source and play an important role in local economies, particularly in the developing world. Unfortunately, in some parts of the world there is a shortage of sand and damaging extractions from beaches, river channels and the seabed are occurring [56].

### (a) Case study: the circular economy

A circular economy (CE) is one that tries to retain materials within the system. It, therefore, attempts to minimize the use of new resources through more efficient processes, prolonging use, reuse, recycling, etc. This is in contrast to a linear or throughput economy that uses virgin resources to manufacture goods which are then disposed of at the end of their life and is, therefore, resource intensive and wasteful. De Wit *et al*. [57], in their 2020 *Circularity Gap Report* showed that about 100 billion tonnes of resources enter the global economy each year, 75% of which are geomaterials, but only 8.6% is recycled. This explains the interest in trying to make the economies of the world more circular in approach so that future generations have access to the same resources we use today. And for some materials recycling has been successful. According to the International Aluminium Institute, 75% of all the aluminium ever produced is still in use today.

The UK is currently very dependent on imports of critical metals such as lithium, cobalt, tin, tungsten and rare earth elements used in modern technologies including electric vehicles, solar photovoltaics, wind farms, etc. [58]. As a result, the project Met4Tech (www.met4tech.org/) has been set up to try to use these technology materials more sustainably by examining how they flow through the economy. The project brings together industry, academics, policy makers, and civic society ‘to deliver research, innovation and the evidence base to move the UK towards a resilient, inclusive, restorative and competitive UK circular economy’ [58, p. 23]. Another key organization is the Ellen MacArthur Foundation which sees CE as designing out waste and pollution, keeping products and materials in use and regenerating natural systems. Important options for geomaterials include choice of ore types, mine design, smelter processes and material recycling. But for all critical minerals, new supplies are still needed so that the focus is currently on making economies more circular rather than fully circular.

## 7. Geotourism and leisure

**Figure 5.**
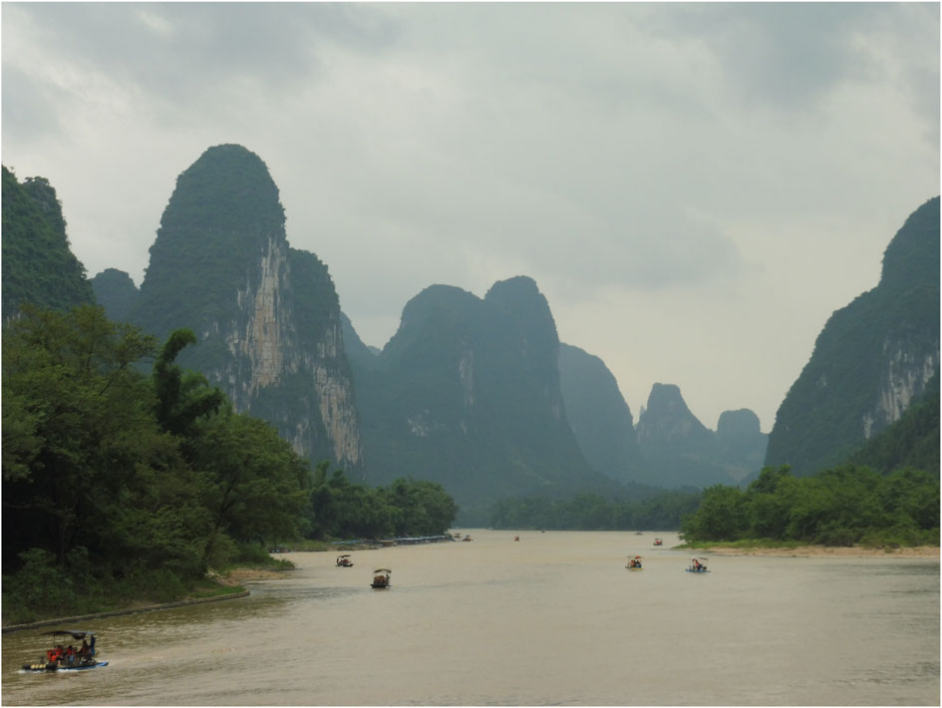
The South China Karst and tourist boats, River Li, China. (Online version in colour.)

Tourism is driven by people's desire to visit new places and experience different cultures. It follows that geotourism is based on geodiversity in that this provides the opportunity to visit different geological and geomorphological places, experience different landscapes and take part in different geoactivities [59,60]. Larwood & Prosser [61, p. 99] believed that ‘Tourists, whether they are aware of it or not, will in some way all be geotourists’, a conclusion that reflects the fact that geomaterials are in use everywhere in historic buildings within urban areas and geology/geomorphology occurs in the wider countryside where tourists are drawn to spectacular rock outcrops, landscapes and dynamic geoprocesses. Geotourism is increasingly seen as not being restricted to geoscience settings but sees these as part of a more inclusive experience including architecture, culture and biodiversity [62,63], not least within UNESCO Global Geoparks (see §10 below). These also strongly promote sustainable geotourism whether the activities are active (e.g. fossil collecting) or passive (e.g. admiring coastal scenery). Many sports and other leisure activities are also based on geodiversity, including rock climbing, skiing, cross country running and whitewater rafting, but other sports require a level playing field (e.g. football and rugby) (figure 5).

### (a) Case study: the search for the world's top geotourism destinations

Several attempts have been made in recent years to list the world's top tourism, nature or geological sites. Gray [64] attempted to develop a provisional list of the top geotourism sites in the world using the criteria of visual impact, site quality and integrity, educational potential and tourist accessibility. Additionally, an attempt was made to ensure that there was a reasonable geographical distribution of sites across the planet and that the sites were both internally geodiverse and representative of the world's geodiversity. Ten sites were selected and sorted into an ordered list (table 2). These 10 sites meet the criteria and there is representation from 6 continents—South America, North America, Africa, Asia, Europe and Australia—as well as some marine sites.

**Table 2.** Proposals for the world's top 10 geotourism destinations.

rank	site	country	main geointerest
1	Iguaçu/Iguazu Falls or Victoria Falls	Brazil/Argentina Zimbabwe/Zambia	waterfalls
2	Grand Canyon	USA	canyon/straigraphy
3	Great Barrier Reef	Australia	coral reef
4	South China Karst to Ha Long Bay	China/Vietnam	karst
5	Yellowstone National Park or Hawaii Volcanoes National Park	USA	geothermal/volcanoes
6	Uluru	Australia	sandstone mount/folklore
7	Central Swiss Alps	Switzerland	mountain/glacial
8	Cal Orcko Parque Cretácico	Bolivia	dinosaurs
9	‘Golden Circle’ Route	Iceland	tectonics/waterfall
10	Table Mountain, Cape Town	South Africa	city physical setting
	or Rio de Janeiro	Brazil	

## 8. Geoheritage

**Figure 6.**
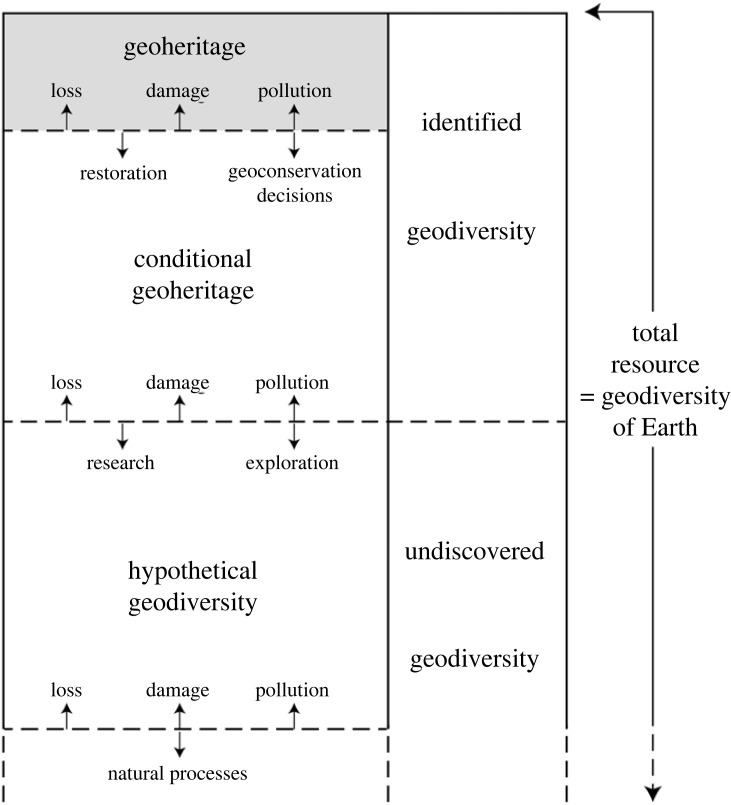
Modified McKelvey Box showing the relationships between geodiversity and geoheritage and between discovered and undiscovered geodiversity (with permission of Elsevier).

The terms ‘geoheritage’ and ‘geodiversity’ are often used interchangeably, but they are in fact different. Sharples [65] was the first to define geoheritage as comprising ‘…those elements of natural geodiversity which are of significant value…’. As figure 6 shows, geoheritage is, therefore, part of the much wider concept of geodiversity. Geoheritage can be lost or damaged due to a large number of human actions but it can also be gained by restoration or by making decisions to conserve more geosites. This is why the area below on figure 6 is named ‘conditional geoheritage’ in that it is conditional on geoconservation decisions. So, the line is dashed to indicate that it can move up or down. Therefore, geoheritage is a value-laden term, in that it depends on geoconservation decisions, whereas geodiversity is a value-free concept, in the sense that it simply a description of abiotic nature. Together, geoheritage and conditional geoheritage constitute the identified geodiversity, but its lower boundary can also move up, if it is lost or damaged, or down through research and exploration. Hypothetical geodiversity is geodiversity yet to be discovered, and this can very easily be lost or damaged as it is unidentified and therefore unrecorded and unvalued. But its lower boundary can also move down by natural processes creating new elements of geodiversity as has been going on since the origin of the Earth 4.6 billion years ago [9,66].

### (a) Case study: landscape restoration

**Figure 7.**
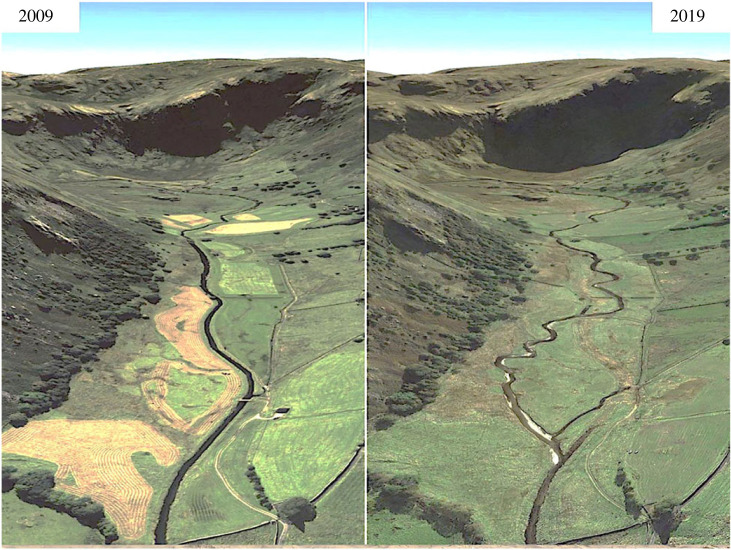
Before and after photos of the remeandered Swindale Beck in Cumbria, England showing the gravel bars, etc. (Google Earth/BBC). (Online version in colour.)

Some confusion sometimes takes place over whether geodiversity can be restored. It is true that bedrock or sediment geosites cannot be restored or rebuilt if they are lost or damaged. They are non-renewable and once they are gone, they are gone. But some other elements of geoheritage and geodiversity can be restored. For example, a very active field of georestoration is river restoration, in which dams, weirs, straightened reaches, concrete channels etc. are removed and rivers allowed to operate naturally once again [66]. Even if human-designed meanders are built into a scheme, in time the river will evolve to a natural channel form. River restoration brings both geodiversity and biodiversity benefits as illustrated by Swindale Beck in Cumbria, England where salmon and trout have returned to spawn in the redeposited gravels in the remeandered reaches where river flows have been slowed (figure 7).

Other fields in which physical landscape restoration takes place include quarry or pit restoration once quarrying or mining has ceased [67]. Erikstad *et al*. [68] describe the landscape restoration of the former Svea coal mines in Svalbard, Norway with the aim of enabling dynamic geomorphological and ecological processes to operate. The project involved the removal of roads, houses, industrial facilities, airports, landfills and quarries, but preservation of heritage features including pre-1946 buildings, structures and mining traces.

Coastal restoration and managed realignment of coasts is also taking place, as is the clearance of vegetation to expose geological sections (e.g. the ‘Purple Horizons’ project in the West Midlands, England) or geomorphological features (e.g. the ‘Back to Purple’ project in Shropshire, England) in which conifers were removed to reveal previously hidden quartzite tors. Finally, the restoration of drained upland bogs and wetlands is taking place in England by blocking channels and ditches in order to raise water levels, restore the bogs to more natural conditions, and reduce flood risk downstream. Remote sensing is being used by Natural England as a more efficient and cheaper way than field survey in which to identify which channels remain to be blocked. These examples of georestoration are important in contributing to nature recovery and rewilding programmes.

## 9. National geoconservation

Geonservation involves activities aimed at protecting geodiversity and geoheritage [65,69]. Several publications review the principles and practice of geoconservation (e.g. [67,70–72]). There are many methods that can be involved [9], but the most common, and often most successful of these, is the protected area approach. In this, boundaries are drawn around the sites to be protected and then given policies and/or legal protection that do not apply outside these boundaries. The question then is to consider how these protected areas are selected. Brilha [73] explains the methodologies involved in the inventorying and quantitative assessment of geosites. In doing this, many countries/regions/organizations have based their selection of sites partly or mainly to represent the geodiversity of the area. For example, in Ireland the Irish Geological Heritage Programme (IGHP) has been aimed at identifying and designating sites ‘to represent the country's geology’ [74]. The IGHP is, therefore, explicitly designed to protect sites to represent the geodiversity of Ireland. Other countries follow a similar approach. For example, Spain's *Natural Heritage and Biodiversity Act* (2007) defines the concept of geodiversity and requires the Spanish Ministry of Environment, regional governments and scientific institutions to maintain an inventory of sites representative of Spanish geodiversity. Similarly, Norway's *Nature Diversity Act* (2009) aims to promote conservation and sustainable use of the full range of habitat and landscape types. This means that geological and landscape diversity are officially recognized alongside biodiversity and that information about their spatial distribution must be researched and mapped for successful implementation of the Act. The UK has not pursued such an integrated approach to nature conservation, but it does have long-standing legal protection for sites of special scientific interest (SSSIs), over 2000 of which are designated for their geoscience interest. This represents about 30% of the total number of SSSIs, the remainder being ecological sites. The aim was to have a network of geosites to ‘reflect the range and diversity of Great Britain's Earth heritage’ [75, p. 45]. In designating new National Park units and National Natural Landmarks, and in trying to avoid duplication of types of site, the USA is partly attempting to conserve sites representative of the country's geodiversity. And in New Zealand, the objective of geoconservation is ‘to ensure the survival of the best representative examples of the broad diversity of New Zealand's geologic features, landforms, soil sites and active physical processes…’ (www.geomarine.or.nz). All these national examples illustrate that these countries, and others not described here, have been selecting geoheritage sites to represent their geodiversity.

### (a) Case study: land-use planning and the Trump international golf course, Foveran Links SSSI, Scotland

Land-use planning systems and laws should be a frontline tool in protecting nature conservation interests, including geoheritage sites, and preventing development that would have a detrimental impact on them. Unfortunately, this is not always the case, either because the law is ignored, or economic/social factors are given priority over environmental ones. This case study is an example of the latter.

In 2006 Trump International Golf Course Scotland Ltd applied for outline consent to build two golf courses on the coastline north of Aberdeen in northeastern Scotland. One of these was to be a championship standard course. Controversially, the layout of this course took in the southern third of the Foveran Links SSSI, a protected coastal sand dune area established in 1984 for both its ecology and geomorphology. The citation states: ‘Foveran Links contain extensive areas of mobile foreshore and sand dunes as well as fixed dunes, dune pasture, marshes and heath’. As well as the golf course, the proposals included:

• clubhouse, golf academy, driving range, practice ground, ancillary buildings, etc.;
• 450-room resort hotel on 8 floors, conference centre and spa;
• 950 holiday apartments in 4 blocks;
• 36 golf villas and 500 houses;
• accommodation for 400 staff;
• a new access road, gatehouse and car parks.

It was estimated that these developments would create about 4700 construction jobs and 1237 full time equivalent (FTE) jobs for ongoing operations. The application was controversial from the start with many written objections, several newspaper articles and television reports. After consideration at two meetings of Aberdeenshire Council, the application was ‘called-in’ by the Scottish government for it to make the final decision. A four-week public inquiry was held in July 2008 in front of three independent inspectors who were to report their findings to the Scottish government. The objectors included Scottish Natural Heritage (SNH), the Scottish government's advisory body on nature conservation. SNH commissioned Dr Jim Hansom, a coastal geomorphologist at Glasgow University, to write a report on the geomorphological interest of the SSSI. This concluded that the area is ‘nationally (and probably internationally) unique on account of the scale and dynamism of the sand sheet’. This is an area of mobile bare sand over 600 m long and 400 m wide that had remained active for most of the previous century. He gave evidence at the inquiry: ‘the proposal to stabilize most of the bare sand surfaces would serve to remove the key scientific interest’. Objectors proposed that the course could be partly resited away from the most important part of the SSSI. Donald Trump himself gave evidence at the inquiry and stated that if the championship course was moved away from the high dunes and mobile sand areas it would no longer be the truly great course he intended. If he was refused permission, he would withdraw the project and the area would lose the investment.

**Figure 8.**
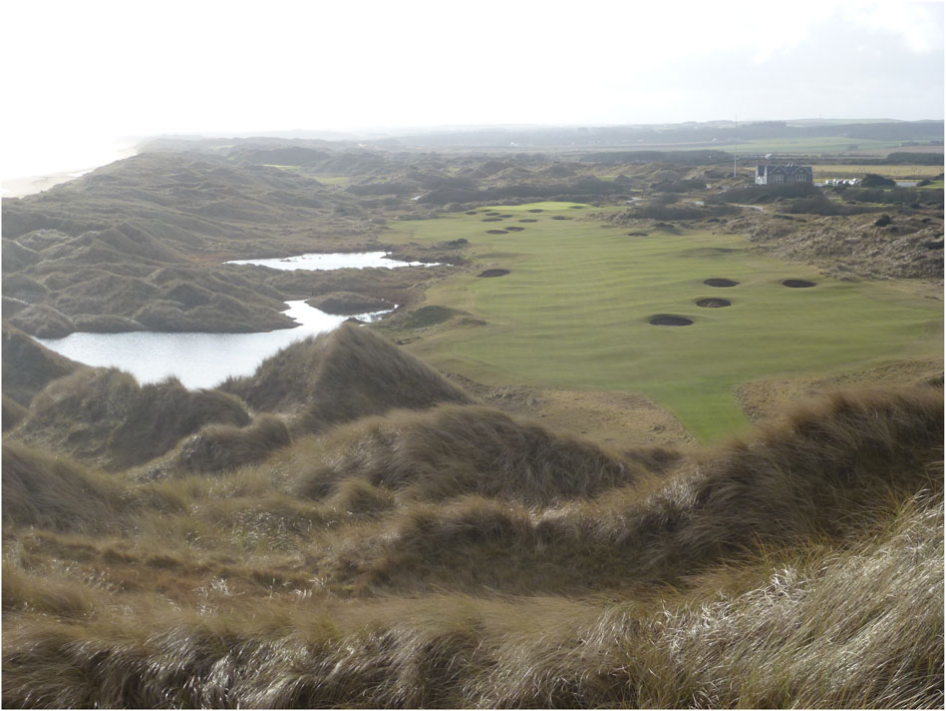
The 18th hole at Trump International Golf Links, Aberdeenshire, Scotland. (Online version in colour.)

In October 2008, the planning inspectors concluded that construction of the course would mean much, though not all, of the geomorphological interest of the SSSI would be compromised as would its overall integrity. They believed that the loss of this dynamism could not be mitigated against. However, their overall conclusion was that the adverse effects were outweighed by the social and economic benefits that were of national, regional and local significance. Scottish government ministers agreed, and the project was given planning consent with 46 conditions. The course was opened in 2012 (figure 8), but so far most of the proposed buildings have not materialized apart from the clubhouse and Menie House, renovated as a 16-bed boutique hotel. The site employs under 100 FTE staff. The second course, outside the SSSI, was given planning consent in 2019, together with 550 houses.

In June 2019, SNH announced that it proposed to denotify the area of the SSSI affected by the golf course as it had ‘adversely affected the coastal geomorphology of Scotland…as well as interrupting natural dune processes'. It, therefore, no longer warranted protected area status and on 9 December 2020 the area was denotified.

This case study is of concern as it demonstrates clearly that environmental damage to protected areas can be tolerated by decision-makers if the social and economic benefits are big enough. But in this case the project was consented without safeguards or conditions to ensure that these benefits were actually delivered. The result is that most of the economic and social benefits have not accrued but the environmental damage has. In my view, the planning decision should have promoted a phased approach in which many more of the social and economic benefits had to be achieved before the golf course became fully operational.

## 10. World Heritage Sites and Global Geoparks

Although there are some other international, geoconservation initiatives (e.g. Global Statotypes Section and Points (GSSPs) and Key Geoheritage Areas (KGAs)), there are two main UNESCO site networks involving geoconservation: World Heritage Sites (WHSs) and Global Geoparks (UGGps). However, neither of these gives legal protection to the sites/areas as this is the responsibility of the country/region in which they lie. But they are important in providing an internationally accepted status that can also be incorporated into national/regional policies and planning [76].

A key point about World Heritage Sites is that they must have ‘outstanding universal value’ which means that they should have ‘significance that is so exceptional as to transcend national boundaries and to be of common importance for present and future generations of all humanity’. An initial stage is for countries to submit a ‘Tentative List’ of sites that they intend to consider nominating for World Heritage status over a 5- to 10-year period. To date, the Convention has been ratified by 194 countries and of these, 185 have produced Tentative Lists. There are *Operational Guidelines* that give details of the application procedures, management requirements and criteria for selection of which there are 10. Six of these refer to cultural sites, and two each covering geoscience and ecology. For geoscience, criterion vii covers ‘natural phenomena or areas of exceptional natural beauty and aesthetic importance’ while criterion viii refers to ‘outstanding examples representing major stages of earth's history’. All sites on the World Heritage List must have conservation and management measures administered by national and/or regional authorities to ensure that the integrity of the site is maintained or enhanced. As of August 2023, there were 1157 World Heritage sites in 167 countries with 900 of these being cultural sites, 218 being natural sites and 39 are mixed sites. Ninty-three sites have been inscribed under criterion viii and these include two from the United Kingdom—the Dorset and East Devon Coast (also known as the Jurassic Coast) and the Giant's Causeway and Coast in Northern Ireland.

Given the then disproportionate number of World Heritage Sites in the developed world, in 1994 UNESCO launched a Global Strategy to try to make the List ‘representative, balanced and credible’. In relation to this, Dingwall *et al*. [77] analysed whether the World Heritage List at that time was representative of the geological column and concluded that many gaps existed, including the absence of any Silurian sites. They also identified 13 geothemes or types of geological/geomorphological features. This was subsequently supplemented by thematic studies of karst and caves [78], volcanoes [79,80] and desert landscapes [81]. All of these have concluded that important gaps exist in the List. McKeever & Narbonne [82] have produced an updated version of the [77] report, reducing the number of geothemes to 11. This important report also discusses how well each theme is currently represented on the World Heritage List and identifies the most significant gaps, e.g. no fossil sites in the Ordovician, Silurian or Permian. So once more, the importance of trying to ensure that the List is representative of the geodiversity of the planet is explicitly recognized.

The second UNESCO international geoscience network comprises Global Geoparks. These are unified areas of land that demonstrate internationally important geoscience and that conserve this geoheritage, improve public understanding of the geosciences and promote regional economic development often through geotourism. They should always involve local communities taking ownership of their geoheritage by protecting it, promoting it and in doing so, gaining some sustainable economic benefit from it. In some cases, the international interest is related to a limited geological or geomorphological theme where most geosites in the geopark are related to this single theme. An example is Vulkaneifel UGGp in Germany, but even here the geopark displays a diversity of maar-craters. However, most geoparks, particularly in Europe, aim to attract visitors by promoting their geodiversity. The clearest example is the Gea Norvegica UGGp southwest of Oslo in Norway, which describes itself as having ‘unique’ and ‘extreme’ geodiversity. ‘Textbook examples can be found in almost any field of geology’ [83]. Geodiversity is, therefore, a major way in which many geoparks promote themselves to the public.

Unlike World Heritage Sites, where the status is permanent except in the few cases where sites have been expelled, Global Geopark status only lasts 4 years after which, there is a revalidation process which can result in membership being lost or renewed for only 2 years to allow issues to be addressed. The first geoparks were established in 2001 but in 2004, 17 European and 8 Chinese geoparks came together to form the Global Geoparks Network (GGN). Then, in 2015, the network was fully adopted as a UNESCO Global Geoparks network (UGGps). As of August 2023, there were 195 Global Geoparks in 48 countries. The Geopark story is one of remarkable success in drawing public attention to the place of geology and geoscience within the wider environment and local culture. The UNESCO Global Geopark network is only likely to expand and flourish in the future because of the perceived benefits that this status brings.

**Figure 9.**
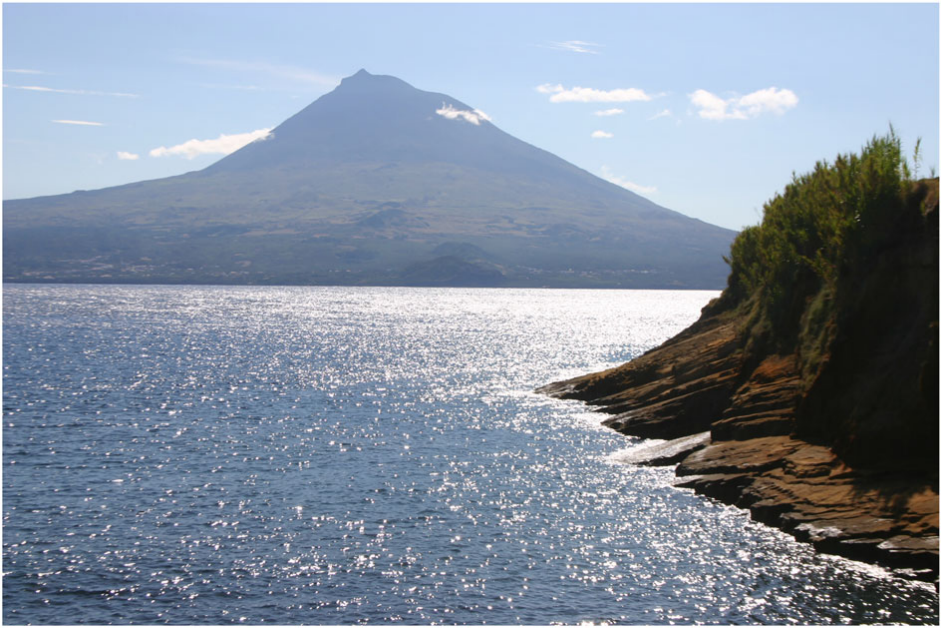
The island of Pico taken from Faial in the Azores UGGp, Portugal. (Online version in colour.)

### (a) Case study: geodiversity of the Azores UGGp, Portugal

The Azores archipelago, a Portuguese Autonomous Region, is part of the Mid-Atlantic Ridge, lying about 1800 km west of mainland Portugal. It is formed of nine main volcanic islands (figure 9) dispersed along a 600 km, WNW–ESE belt at the triple junction of the Eurasian, North American and African tectonic plates. Not surprisingly, therefore, the islands are highly tectonically and volcanically active and this is the key to their geodiversity, as described by Nunes [84]. There have been 26 eruptions recorded since the start of human occupation in the early fifteenth century, and several magnitude 7 earthquakes that have caused 6350 deaths and produced well-developed fault scarps. The islands comprise 27 main volcanic systems with 16 main central volcanoes (most of them silicic and with summit subsidence calderas), and 11 volcanic ridges associated with basaltic fissure volcanism. In total, nine major polygentic volcanoes and seven fissures are classed as active though currently dormant. Offshore there are several active, submarine ridges and banks, one of which is the most recent eruption in 1998–2001. In addition, there are about 1750 monogenetic volcanoes, including domes, tuff rings, maars, scoria and spatter cones, and eruptive fissures. Other volcanic structures include hydrothermal fields, pillow lavas, columnar jointing, lava tubes, aa and pahoehoe lavas, pyroclastic deposits, fault scarps, fossiliferous marine deposits (Miocene–Quaternary) and fajas (littoral platforms of volcanic or landslide origin). This geodiversity has been recognized as a natural laboratory of international importance for active volcanism, volcanic and tectonic landforms and features, global plate tectonics and neotectonics [84], and brought it UGGp status for the quality and diversity of its geology and geomorphology in 2015, including 121 geosites [85].

## 11. Sustainability

The relevance of geodiversity to sustainability can be illustrated by the potential input that geodiversity has to the UN's Sustainable Development Goals (SDGs). These 17 goals and 169 targets were adopted by the UN in 2015 with the aim of achieving them within 15 years, i.e. by 2030. But the goals are very ambitious as they are aimed at ending poverty and hunger, facilitating sustainable economic growth and protecting the environment. The geosciences were not involved in developing the goals, yet it is very clear that progress towards achieving them is more likely if geoscience knowledge is integrated into the processes. In particular, geodiversity and geosystem services thinking will be crucial to achieving advances [86,87]. Also relevant in this regard is the poster produced by the Geological Society of London that demonstrates how the many geoscience subdisciplines relate to the SDGs (https://www.geolsoc.org.uk/~/media/shared/documents/education%20and%20careers/Resources/Posters/Geoscience%20for%20the%20Future%20poster.pdf?la=en). A further development is the emergence of essential geodiversity variables (EGVs) [88] to complete the four major Essential Variables that help us to identify, measure and monitor environmental change on the planet. The other three relate to Biodiversity (EBVs: 2013), Climate (ECVs: 2014) and Oceans (EOVs: 2016).

### (a) Case study: geodiversity and sustainability at the Xitle Volcano, Mexico City

Xitle comprises a scoria cone, 140 m high and 500 m wide, and an 80 km^2^ lava field on which 600 000 people in Mexico City now live. It erupted once 1700 years ago and now lies in the southern part of the city. According to Guilbaud *et al*. [89, p. 1], the cone and lavas have ‘significant geodiversity’ and associated pedodiversity and biodiversity. In particular, the lavas are very well exposed due to thin soils and extensive quarrying. They describe four geosites, assess their value, and discuss their potential for addressing issues such as nature conservation, environmental sustainability, social inequalities and natural hazards. The rapid growth of Mexico City in recent decades means that much of the natural environment has been destroyed so that it is even more important to save the geodiversity that creates a wide range of microhabitats for a considerable number of species [SDG15]. The geosites are ‘key locations for educating on sustainability and natural hazards [SDG4]…They can be used to bring people to understand the relationship between volcanic and human activity, conserve and value key elements of the city's identity [SDG11], improve human well-being [SDG3], help lower social inequalities [SDG10], and raise awareness on risks [SDG11]’ [89, p. 24]. As such, this study illustrates how geoscience can contribute to many of the UN's SDGs.

## 12. Conclusion

These 10 major topics demonstrate how geodiversity has flourished over the last 30 years and led to several new research fields. The 10 case studies give a deeper insight into how the research has evolved in significant ways. It might even be regarded as a geoscientific paradigm in that it meets various definitions of ‘paradigm’. Thomas Kuhn [90] argued that ‘normal science’, which advances by gradual accumulation of evidence, can be interrupted by scientific revolutions or paradigm shifts in which new ways of thinking or new views of reality are introduced, accepted by an intellectual discipline, and allow significant advances to be made within the subject. In my view, the evolution of geodiversity over the last 30 years accords with this description. Dictionary definitions of ‘paradigm’ include:

‘a philosophical and theoretical framework of a scientific school or discipline within which theories, laws, and generalizations and the experiments performed in support of them are formulated’ (Merriam-Webster); ‘a theory or a group of ideas about how something should be done, made, or thought about’ (Britannica); ‘a set of assumptions, concepts, values, and practices that constitutes a way of viewing reality for the community that shares them, especially in an intellectual discipline’ (American Heritage).

The Oxford English Dictionary defines a paradigm shift as ‘a great and important change in the way something is done or thought about’. In my view, given how geodiversity has become accepted internationally, advanced our understanding of the world, and demonstrated how society benefits from living on a geodiverse planet since its first use in 1993, it clearly today constitutes a significant, geoscientific paradigm.

## Data Availability

This article has no additional data.
